# Phylogeography of the reticulated python (*Malayopython reticulatus* ssp.): Conservation implications for the worlds’ most traded snake species

**DOI:** 10.1371/journal.pone.0182049

**Published:** 2017-08-17

**Authors:** Gillian Murray-Dickson, Muhammad Ghazali, Rob Ogden, Rafe Brown, Mark Auliya

**Affiliations:** 1 Royal Zoological Society of Scotland (RZSS) WildGenes Laboratory, Edinburgh, United Kingdom; 2 Trace Wildlife Forensics Network, Edinburgh, United Kingdom; 3 KU Biodiversity Institute, 1345 Jayhawk Blvd, Dyche, Lawrence, KS, United States of America; 4 Helmholtz Centre for Environmental Research – UFZ, Leipzig, Germany; National Cheng Kung University, TAIWAN

## Abstract

As an important economic natural resource in Southeast Asia, reticulated pythons (*Malayopython reticulatus* ssp.) are primarily harvested from the wild for their skins—which are prized in the luxury leather goods industry. Trade dynamics of this CITES Appendix II listed species are complex and management approaches on the country or regional level appear obscure. Little is known about the actual geographic point-of-harvest of snakes, how genetic diversity is partitioned across the species range, how current harvest levels may affect the genetic viability of populations, and whether genetic structure could (or should) be accounted for when managing harvest quotas. As an initial survey, we use mitochondrial sequence data to define the broad-scale geographic structure of genetic diversity across a significant portion of the reticulated python’s native range. Preliminary results reveal: (1) prominent phylogenetic structure across populations east and west of Huxley’s modification of Wallace’s line. Thirty-four haplotypes were apportioned across two geographically distinct groups, estimated to be moderately (5.2%); (2) Philippine, Bornean and Sulawesian populations appear to cluster distinctly; (3) individuals from Ambon Island suggest recent human introduction. *Malayopython reticulatus* is currently managed as a single taxonomic unit across Southeast Asia yet these initial results may justify special management considerations of the Philippine populations as a phylogenetically distinct unit, that warrants further examination. In Indonesia, genetic structure does not conform tightly to political boundaries and therefore we advocate the precautionary designation and use of Evolutionary Significant Units within *Malayopython reticulatus*, to inform and guide regional adaptive management plans.

## Introduction

Habitat loss and degradation as a result of unsustainable per capita consumption of natural resources and rising human population levels [1] are having detrimental impacts on ecosystems and biodiversity, particularly in developing countries [2–4]. Overexploitations of natural resources and agricultural activity have been identified as some of the most prominent threats to biodiversity [5]. Unsustainable exploitation of wildlife [6], particularly reptiles [7,8], has been increasingly reported in Southeast Asia [9–13].

Human and pythons have a lengthy historical (and potentially evolutionary) association [14]. The international and commercial trade in python skins can be traced back to between 1910 [15] and the 1920s [16–18] with contemporary uses for python-derived products spanning the fashion, food and traditional medicine industries [19,20]. Trade in reptile skin and leather products is valued at $339 million, approximately 5% of the legal, global wildlife trade [21], with five Southeast Asian python species (*Malayopython reticulatus* ssp., *Python bivittatus* ssp., *P*. *curtus*, *P*. *brongersmai* and *P*. *breitensteini*) being heavily exploited for this purpose. Among these the reticulated python (*M*. *reticulatus* ssp.) is the most economically important species [18] with approximately 350,000 skins legally exported annually for the high-end fashion market alone [20].

Formerly recognized in the genus *Python*, the reticulated python was genetically and morphologically allocated as a distinct clade, together with the Lesser Sunda python (*Python timoriensis*), and thereinafter included in the genus *Broghammerus* [22,23]. However, this genus [24] is considered invalid as it lacked accompanying data and analysis [25]. Reynolds et al. [26] therefore ascribed the new genus *Malayopython*, an action that has since been supported [27,28] and includes the two species *M*. *reticulatus* and *M*. *timoriensis*.

**Fig 1:**
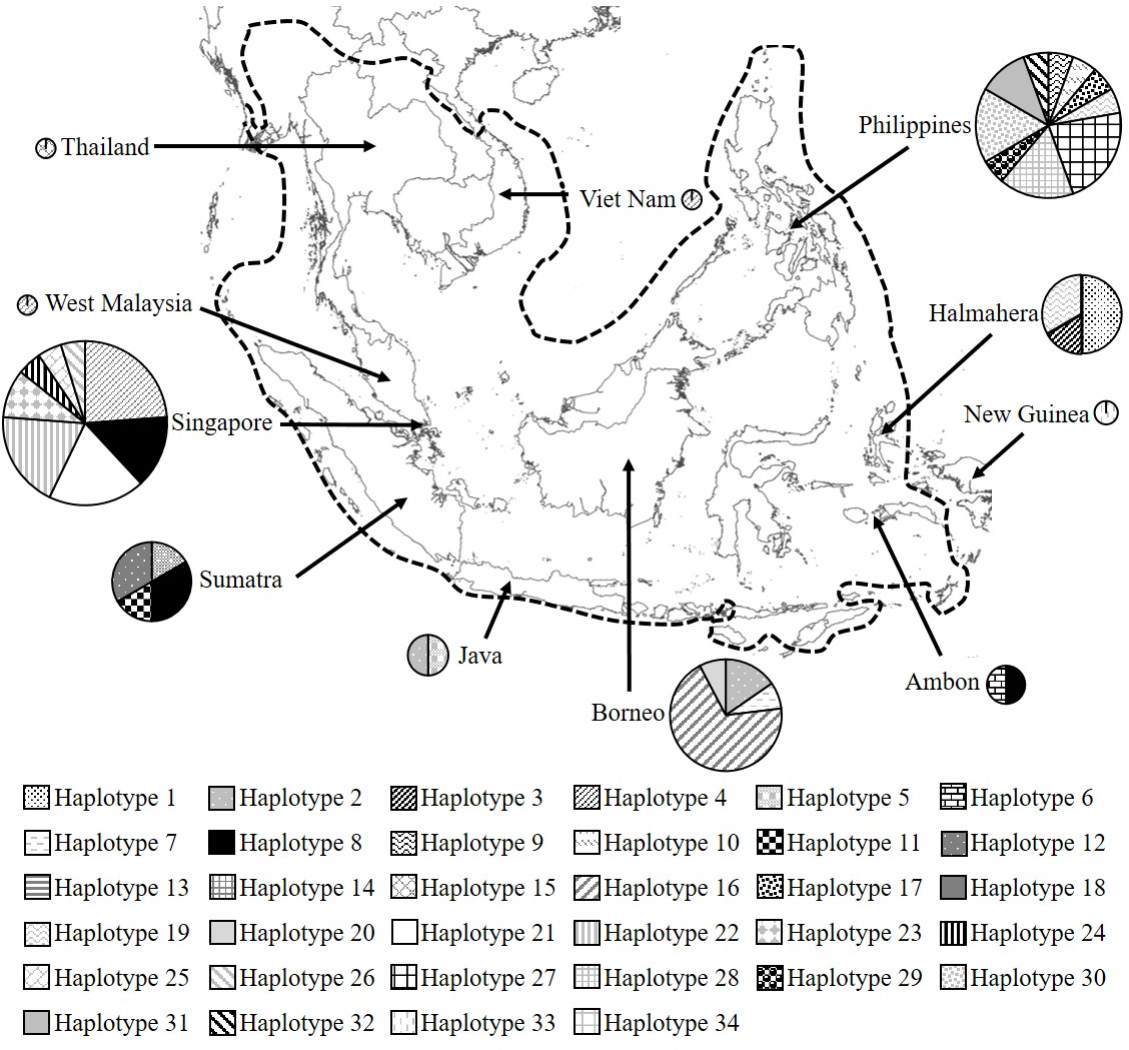
Sample geographic origins. Distribution of the reticulated python across Southeast Asia, as indicated by the dotted line (after [3]). Pie chart size indicates the number of samples sequenced from each location and segments represent the frequency of each mitochondrial haplotype resolved.

The present global range of *Malayopython reticulatus* is explained by the combination of the complex, regional geological history (particularly that of insular Southeast Asia) [29], the species’ excellent dispersal ability [30], and human introductions [31]. The species is distributed extensively across continental and insular Southeast Asia (Fig 1) with distinct insular ‘morphs’ (colour patterns) from Indonesia recognised in the pet and skin trade [32,33]. Although taxonomic subdivisions have not been extensively applied across the broader species range, morphologically and genetically differentiated populations (*M*. *r*. *saputrai* and *M*. *r*. *jampeanus*) have been identified on Selayar and Tanahjampea Island, respectively [34]. Phenotypic distinctiveness of the Philippines population has also been noted (Auliya and Brown, *pers*. *obs*.).

All python species are listed on Appendix II of the Convention on International Trade of Endangered Species of Fauna and Flora (CITES) [35], except for the Indian python (*Python molurus)* which is listed on CITES Appendix I. Appendix II listing allows commercial trade that is regulated through a permitting process [36]. Data and trends in the trade of *Malayopython reticulatus* skins, compiled during a CITES commissioned study to examine commercial trade and review the current taxonomic status, distribution, biology and ecology in Asian pythons’, identified Indonesia as the primary country supplying skins of *M*. *reticulatus* to the global market [18]. With export permits documenting approximately 700,000 skins in 1987/1988, the harvest quota of *M*. *reticulatus* in Indonesia was limited to 445,000 specimens in 1989. However, the number of skins documented on export permits from Indonesia totalled 555,882 for the same year and therefore traceability issues (origin of skins and trade routes) were already being called to interest [18]. Trade of wild pythons was banned in Thailand in 1992 [Wild Animal Reservation and Protection Act, B.E. 2535 (A.D. 1992); www.ThaiLaws.com], the Philippine governments banned the export of *M*. *reticulatus* in 1986, and Viet Nam banned wild harvest of *M*. *reticulatus* in 1998 [37]. Today, Malaysia and Indonesia are the major countries of origin and export for skins of wild *M*. *reticulatus* [20] while the only country that claims to regularly export captive-bred specimens for the skin trade is Viet Nam. During the period 2000 and 2013, the country exported 776,916 skins and 380,870 metres of skins of *M*. *reticulatus* that were declared as captive-bred (CITES Trade Database—http://trade.cites.org).

Although harvest levels vary across regions, significant declines of the species have been reported in Bangladesh, Indonesia, Lao PDR, Malaysia, Myanmar and the Philippines–all of which has brought in to question the sustainability and legality of intense, large-scale commercial trade in *M*. *reticulatus* skins [18]. Whilst traders in Indonesia may not perceive a depletion of local populations, this may be explained by the increase in harvest areas over years, to fetch the annually allocated quotas [3]. Hunting areas have increased in West Kalimantan (province of Indonesian Borneo) as a result of a growing trade system and the improved mobility of hunters, but may also be caused by a local decline in populations due to the expansion of agricultural land [3]. Sustainability of annual harvest levels are also debatable, due to the pressures of continuous forest loss in that geographic realm [38].

When combined with the high rate of population growth in these areas (with the exception of Thailand, it is estimated at 1.17% above the world mean average in 2010) [39] and associated use of natural resources, it is unknown how current levels of harvesting will affect the viability of *M*. *reticulatus* populations throughout the many political and geographic regions of its distribution. Furthermore, little is known about how levels of genetic diversity are partitioned across the species range, nor how variable harvest rates will affect local population viability. The question of python population sustainability has prompted industrial stakeholders to identify a need for traceability systems for proving the legal origin of python skins in trade, ideally to the point of harvest [40,41].

Examples of DNA-based tools to inform monitoring and enforcement are widespread. They have been used effectively during wildlife forensic investigations (see [42] for review)—to identify parts of animal derivatives in an otherwise unidentifiable sample. Forensic procedures have been developed to identify animal parts or derivatives in the traditional Chinese medicine trade (e.g., snakes: [43,44]), identify turtle species from samples of shell [45], to verify origin [46] either where illegal activity or fraudulent claims are suspected e.g., *Testudo graeca* [47] and *Morelia boeleni* [48], and to authenticate population of origin where regional differences in population viability, management or harvest quotas exist (e.g., ivory, [49]; shark fins, [50]; regulated fisheries, [51]). Taxonomic clarification prior to monitoring trade was suggested for short-tailed pythons, the polytypic species *Python curtus* [52]. High levels of divergence were found between the three, recognised subspecies, all of which were being exploited as a single taxonomic unit across Borneo and Sumatra. The authors suggested elevating each taxon to species level, with recommendations that they no longer be managed as a single biological taxon. As with *Python curtus* ssp., consideration of genetic structure in establishing management plans has been recommended for populations of the intensely traded Nile monitor lizard (*Varanus niloticus*) [53,54], to avoid unnecessary erosion of individual genetic partitions [55]. Despite recognising *Malayopython reticulatus* as an important commercial resource across much of its native range, little consideration has been given to examining the distribution of genetic diversity, much less to incorporating the data into regional management plans.

The legality of wild-harvest to meet the demands of the python skin trade, and the existence of registered python farms, varies among countries [37,56,57]. Where wild-harvest is not permitted (i.e. Cambodia, Thailand, Viet Nam) but laundering is suspected, cross-border trade activities and confiscations have been reported [40,58–62], suggesting that a portion of ‘captive-bred’ specimens may be of wild origin. In such instances, it would be beneficial to be able to identify either the farmed or wild source of a sample. However, to use genetic data as evidence for enforcement would require farmed individuals to be genetically distinguishable from their wild counterparts, or the establishment of a verifiable breeding registration scheme. As official python farms only exist across a small portion of the species range and farming practices vary considerably across the industry [56], it is unlikely that the level of genetic differentiation would be uniform for all farmed populations. Development of a DNA-based traceability tool would therefore require scrutiny on a farm-by-farm basis to assess its feasibility in each case. Alternatively, if wild populations are genetically structured across distinct geographic areas, genetic data can be used to trace the geographic origin of an individual and from this, determine whether its geographic origin was the same as reported on the associated permit [11,12]. Where wild-harvest is permitted, extensive testing of python skin shipments would facilitate assessment of whether quota levels were being adequately complied with, monitored and adapted. This information could also be used to help identify potential situations where wild caught (e.g. live reptile) individuals are fraudulently labelled as captive bred [7,63].

To enable development of DNA-based traceability tools it is first necessary to understand whether population genetic structure exists across the species range, and assess whether the genetic structure reflects geographic boundaries or partitions, whilst quantifying differences that exist across known localities. This approach provides reference data with which to compare future samples of ‘unknown origin’, and an assessment of the distribution of genetic diversity will help direct future taxonomic investigation across different regions [55,64,65]. This preliminary approach may also be useful for defining and devising an adaptive management strategy for *Malayopython reticulatus*. It should be noted however that access to comprehensive reference sample sets can be challenging. When sub-optimal, comparative observations such as morphological comparison (see [34]) are not possible and results remain provisional. To this end we present mitochondrial sequence data derived from a collection of historical and contemporary samples of *M*. *reticulatus* to investigate broad-scale geographic structure of genetic diversity across a significant portion of the species native range. Provisional genetic structure is examined with respect to biogeographical variability across the Indo-Australian archipelago, and the feasibility of using these data for identifying the geographic origin of individual samples is assessed. We identify distinct genetic lineages as candidate targets for conservation (such as Evolutionary Significant Units [66]), to facilitate and guide further fine-scale analysis to improve the resolution of potential traceability tools for conservation management and enforcement of laws relevant to illegal trade.

## Materials and methods

Handling of live animals for samples collected in the Philippines was approved under the University of Kansas’ IACUC authorisation 158–04.

### Sampling and DNA extraction

Eighty-nine *Malayopython reticulatus* samples were used in this study, representing populations from Thailand and Viet Nam, West Malaysia, Singapore, Sumatra, Java, Lesser Sundas, Borneo, Ambon, Halmahera and the Philippines (see Fig 1). Samples of tissue were collated from museum voucher specimens that had been collected across the Southeast Asian distribution of *M*. *reticulatus* between 1862 and 2014 (see S1 Table for details). Blood samples were also provided from the Singapore Zoological Gardens, collected from individual live pythons encountered in and around residential areas. Ventral scale clip samples and shed skins were taken from captive held specimens of known geographic origin. It should be noted that one sample obtained from a museum collection was labelled as ‘New Guinea’, however, the species is not known to naturally occur in New Guinea and so this locality is considered in error. Tissue samples preserved either in ethanol, or in formalin prior to transfer to ethanol, were also frozen at -20°C. Blood samples were stored immediately upon collection in Ethylenediaminetetraacetic acid (EDTA) tubes and subsequently stored at -20°C. Shed skin was dried and then stored at -20°C. Total genomic DNA was extracted from individual blood samples, dried shed skin and museum samples preserved in ethanol using the DNeasy blood and tissue kit (Qiagen Ltd) according to the manufacturer’s protocol. Samples previously in contact with formalin were first soaked overnight in phosphate-buffered saline (PBS) prior to DNA extraction using the QIAamp DNA investigator kit (Qiagen Ltd), including 1 ng carrier RNA per extraction as per the manufacturers protocol.

### Mitochondrial DNA sequencing and analysis

A suite of primers was used to PCR amplify approximately 1200 base pairs (bps) of the mitochondrial cytochrome *b* (Cyt *b*) and adjacent, partial control region. Although regularly used for phylogeographic inference, a duplicated control region is present in the snake mitochondrial genome due to gene rearrangements [67]. This makes it difficult to validate amplified fragments and so primers which target the cytochrome b gene were selected instead [67,68]. Details of primers are given in Table 1. Fragment 1 and Fragment 2 were amplified from fresh tissue, blood and dried skin samples, whereas Fragment 3, Fragment 4 and Fragment 5 were amplified from museum-derived samples. Primers sets Mretic1a, Mretic2 and Mretic3 were designed to produce smaller, overlapping amplicons that could be successfully amplified from fragmented DNA. The PCR amplifications were performed in a total volume of 20 μl using a MJ Research PTC-100 thermal cycler (Waterton, MA). The final reaction mix contained 2 μL (at 10–50 ng/μL) of template DNA, 14 μL Maxima Hot Start PCR mastermix (which includes Maxima Hot Start Taq DNA polymerase, 2X hot start PCR buffer, 0.4 mM of each dNTP, 4 mM Mg^2+^) and 2ul of each forward and reverse primer at 10uM. The PCR profile consisted of an initial denaturation step of 5 min at 95°C, 40 cycles of PCR consisting of 30 seconds denaturation at 95°C, 30 seconds of annealing at 50°C and 60 seconds extension at 72°C, ending with 10 mins at 72°C. Products were cleaned using 1 μl of a 1:1 Exo1/FastAP solution (ThermoFisher Scientific) and incubated for 15 mins at 37°C and 15 mins at 85°C. Two microliters of the purified product were prepared for sequencing with BigDye® Terminator v3.1 Cycle Sequencing Kits, as per manufacturers instruction (ThermoFisher Scientific), and sequenced on an ABI3730 capillary sequencer (Edinburgh Genomics GenePool facility, Edinburgh).

**Table 1:** PCR primer sequences.

Fragment	Primer	Sequence (5’–3’)	Fragment size (bps)	Source
1	Snake12L	CAGCCAAYATCAAYCTAGCATTTTCATC	900	[67]
	H15916	GCCCAGCTTTGGTTTACAAGA		
2	L14841	AAAAAGCTTCCATCCAACATCTCAGCATGATGAAA	300	[68]
	H15149	AAACTGCAGCCCCTCAGAATGATATTTGTCCTCA		
3	Mretic1a_F	CACACTAATAGCCACCGCTT	287	*This study*
	Mretic1a_R	TTGTCGATGTCTGGGTTGGT		
4	Mretic2_F	ACACGTTATCTTACTCCACGAAG	326	*This study*
	Mretic2_R	AGCTGTGTGTGTGAATGGGA		
5	Mretic3_F	TCTACGATCCATCCCCAATAAAC	241	*This study*
	Mretic3_R	GGAATGGGATGGAGATGAAGAA		

Table 1 provides details of the primers used to amplify fragments of the mitochondrial cytochrome *b* and adjacent control region from samples of *Malayopython reticulatus*.

Fragments 1 and 4 were sequenced in both the forward and reverse directions using PCR primers, whereas Fragments 2, 3 and 5 were sequenced in the forward direction only. Samples exposed to formalin were sequenced in both directions for all fragments to check for consistency. Sequence chromatograms were checked by eye to ensure unambiguous base-pair determination, and consensus sequences were edited using the trace editor in MEGA version 6 [69]. Fragment sequences were concatenated for each individual and subsequently aligned using CLUSTAL W [70]. The optimal nucleotide substitution model for aligned sequences was identified as TrN+I (proportion of invariable sites = 0.834, gamma = estimated from the data) using BIC as implemented in jModeltest v2.1.10 [71,72].

Hierarchical phylogenetic connectivity between individuals was inferred using both maximum likelihood (ML) and Bayesian approaches. Maximum likelihood inference was parameterised by the defined model and branch support was assessed over 1000 bootstrap replicates using PhyML version 3 [73], implemented within Geneious v 8.0.4 [74]. Bayesian inference was conducted using MrBayes [75] as implemented in Geneious, and analyses were conducted using HKY+I as the closest available substitution model. The model was run for 1,500,000 MCMC iterations after discarding 500,000 burn-in generation, and subsampled every 100 trees across 4 heated chains. The posterior distribution contained a total of 3601 samples, which were summarized by consensus. The resultant phylogenies were rooted with outgroups *Python bivittatus* (NCBI accession number JX401133.1) and *Malayopython timoriensis* (NCBI accession number EF545106.1). The probability of reciprocal monophyly under the null model of random coalescence [76] was calculated using a ‘Species Delimitation’ plugin, available for implementation in the Geneious software. Haplotype genealogy and geographic distribution were examined using a median joining network, constructed using PopArt software [77], under default parameters. The networks were constructed using both the full sequence data set and for individual amplified fragments (e.g. Fragment 2 only) to check for congruence between the clusters resolved.

Haplotype and nucleotide diversities (including the number of haplotypes, Nei’s haplotype (gene) diversity—H_d_, number of segregating sites, and nucleotide diversity—π) were calculated for each putative island population and each haplotype group using DnaSP ver. 5.10.01 [78]. Haplotype richness was calculated using Contrib software [79] for individual populations only. A hierarchical analysis of molecular variance (AMOVA) was used to estimate genetic structure within and between geographically distinct groups, with deviation from null distribution tested with 1000 permutations. The pairwise fixation index Φ, (analogous to Wrights Fst) under the Tamura-Nei substitution model was calculated in Arlequin v 3.5 [80]. Population genetic analyses were completed using only sampling locations represented by > 6 individuals whereas diversity estimates were made for all island populations. Haplotype sequences were also compared to those available for *M*. *reticulatus* on the NCBI database and permitted comparison across the entire amplified sequence length (NCBI accession numbers U69860.1 and U69859.1).

## Results

Analysis of 830 bps of concatenated Cyt *b* and partial control region sequence revealed 34 unique haplotypes throughout 81 samples of *Malaopython reticulatus*. Excluding sites with missing data, haplotypes were defined by 62 variable positions identified along the remaining 570 base pairs, of which 49 were parsimony informative (Table 2). Sequences are deposited on Genbank (accession numbers: MF576180 –MF576213).

**Table 2:** Variable base pair positions across the cytochrome b and control region fragment.

		Haplotype																																	
		1	2	3	4	5	6	7	8	9	10	11	12	13	14	15	16	17	18	19	20	21	22	23	24	25	26	27	28	29	30	31	32	33	34
**Variable position**	**197**	G	.	A	.	.	.	.	.	.	.	.	.	.	.	.	.	.	.	.	.	.	.	.	.	.	.	.	.	.	.	.	.	.	.
	**205**	T	C	.	C	C	C	C	C	.	C	C	C	C	C	C	C	.	.	.	C	C	C	C	C	C	C	.	.	.	.	.	.	.	.
	**229**	A	G	.	G	.	G	G	G	.	.	G	G	G	.	G	G	.	.	.	G	G	G	G	G	G	G	.	.	.	.	.	.	.	.
	**238**	A	C	.	C	C	C	C	C	.	C	C	C	C	C	C	C	.	C	.	C	C	C	C	C	C	C	.	.	.	.	.	.	.	C
	**241**	A	.	.	.	.	.	.	.	.	.	.	.	.	.	.	.	.	.	.	.	.	.	.	.	.	.	.	G	.	.	.	.	.	.
	**277**	C	T	.	T	T	T	T	T	.	T	T	T	T	T	T	T	.	T	.	T	T	T	T	T	T	T	.	.	.	.	.	.	.	T
	**281**	C	.	.	.	.	.	.	.	.	.	.	.	.	.	.	.	.	T	.	.	.	.	.	.	.	.	.	.	.	.	.	.	.	.
	**286**	T	.	.	.	.	.	.	.	.	.	.	.	.	.	.	.	.	C	.	.	.	.	.	.	.	.	.	.	.	.	.	.	.	C
	**292**	T	.	.	.	.	.	.	.	.	.	.	.	.	.	.	.	.	C	.	.	.	.	.	.	.	.	.	.	.	.	.	.	.	C
	**302**	A	G	G	G	G	G	G	G	G	G	G	G	G	G	G	G	G	G	G	G	G	G	G	G	G	G	G	G	G	G	G	G	.	G
	**304**	A	.	.	G	G	G	.	G	.	G	G	.	G	.	G	G	.	.	.	.	G	G	G	G	G	G	.	.	.	.	.	.	.	.
	**307**	C	T	.	T	.	.	T	T	T	.	T	T	T	.	.	T	T	.	.	T	T	T	T	T	T	T	T	.	.	.	.	.	.	.
	**314**	T	.	.	.	.	.	.	.	.	.	.	.	.	.	.	.	.	C	.	.	.	.	.	.	.	.	.	.	.	.	.	.	.	C
	**331**	T	.	.	.	.	.	.	C	.	.	.	.	.	.	.	.	.	.	.	.	C	C	.	.	.	C	.	.	.	.	.	.	.	.
	**337**	A	.	.	.	.	.	.	.	.	.	.	.	.	.	.	.	.	G	.	.	.	.	.	.	.	.	.	.	.	.	.	.	.	G
	**349**	G	A	.	A	A	A	A	A	.	A	A	A	A	A	A	A	.	A	.	A	A	A	A	.	A	A	.	.	.	.	.	.	.	A
	**367**	G	.	.	A	A	A	.	A	.	A	A	A	A	A	A	.	.	A	.	.	A	A	A	A	A	A	.	.	.	.	.	.	.	A
	**373**	T	C	C	C	C	C	C	C	C	C	C	C	C	C	C	C	C	C	C	C	C	C	C	C	C	C	.	.	.	.	.	.	C	C
	**431**	T	.	.	.	.	.	.	.	.	.	.	.	.	C	.	.	.	C	.	.	.	.	.	.	.	.	.	.	.	.	.	.	.	C
	**440**	T	C	.	.	.	.	.	.	.	.	.	.	.	.	.	.	.	.	.	.	.	.	.	.	.	.	.	.	.	.	.	.	.	.
	**446**	A	G	.	G	.	.	G	G	.	.	G	G	G	.	G	G	.	.	.	G	G	G	G	G	G	G	.	.	.	.	.	.	.	.
	**449**	T	C	.	C	C	C	C	C	.	C	C	C	C	C	C	C	.	C	.	C	C	C	C	C	C	C	.	.	.	.	.	.	.	C
	**453**	T	C	.	C	C	C	C	C	.	C	C	C	C	C	C	C	.	.	.	C	C	C	C	C	C	C	.	.	.	.	.	.	.	.
	**455**	C	.	.	.	.	.	.	.	.	.	.	.	.	T	.	.	.	.	.	.	.	.	.	.	.	.	.	.	.	.	.	.	.	.
	**462**	T	.	.	.	.	.	.	.	.	.	.	.	.	.	.	.	.	C	.	.	.	.	.	.	.	.	.	.	.	.	.	.	.	C
	**487**	T	C	.	C	C	C	C	C	.	C	C	C	C	C	C	C	.	C	.	C	C	C	C	C	C	C	.	.	.	.	.	.	.	C
	**490**	T	.	.	C	.	.	.	C	.	.	.	C	.	.	.	.	.	.	.	.	C	C	C	.	C	C	.	.	.	.	.	.	.	.
	**499**	A	G	.	.	.	.	G	.	.	.	.	.	.	.	.	G	.	.	.	G	.	.	.	.	.	.	.	.	.	.	.	.	.	.
	**505**	C	T	.	T	T	T	T	T	.	T	T	T	T	T	T	T	.	T	.	T	T	T	T	T	T	T	.	.	.	.	.	.	.	T
	**523**	T	C	.	C	C	C	C	C	.	C	C	C	C	C	C	C	.	C	.	C	C	C	C	C	C	C	.	.	.	.	.	.	.	C
	**556**	G	A	.	A	A	A	A	A	.	A	A	A	A	A	A	A	.	.	.	A	A	A	A	A	A	A	.	.	.	.	.	.	.	.
	**563**	T	C	.	C	C	C	C	C	.	C	C	C	C	C	C	C	.	C	.	C	C	C	C	C	C	C	.	.	.	.	.	.	.	C
	**568**	T	C	.	C	C	C	C	C	C	C	C	C	C	C	C	C	.	C	.	C	C	C	C	C	C	C	.	.	.	.	C	.	.	C
	**583**	A	.	.	.	.	.	.	.	.	.	.	.	.	.	.	.	.	.	.	.	.	.	.	G	.	.	.	.	.	.	.	.	.	.
	**592**	T	C	.	C	C	C	C	C	.	C	C	C	C	C	C	C	.	C	.	C	C	C	C	C	C	C	.	.	.	.	.	.	.	C
	**610**	G	A	.	A	A	A	A	A	.	A	A	A	A	A	A	A	.	.	.	A	A	A	.	.	A	A	.	.	.	.	.	.	.	.
	**613**	A	.	.	.	.	.	.	.	.	.	.	.	.	.	.	.	.	G	.	.	.	.	.	.	.	.	.	.	.	.	.	.	.	G
	**620**	T	.	.	.	.	.	.	.	.	.	.	.	.	.	.	.	.	.	.	C	.	.	.	.	.	.	.	.	.	.	C	.	.	.
	**622**	A	.	.	.	G	G	.	.	.	G	.	.	.	.	.	.	.	.	.	.	.	.	.	.	.	.	.	.	.	.	.	.	.	.
	**624**	T	.	.	.	.	.	.	.	.	.	.	.	.	C	.	.	.	.	.	.	.	.	.	.	.	.	.	.	.	.	.	.	.	.
	**625**	A	.	.	.	.	.	.	.	.	.	.	.	.	.	.	.	.	.	.	.	.	.	.	.	.	.	.	.	.	.	G	.	.	.
	**628**	G	A	.	A	A	A	A	A	A	A	A	A	A	A	A	A	A	.	.	A	A	A	A	A	A	A	A	.	.	A	.	.	.	.
	**634**	T	.	.	.	.	.	.	.	.	.	.	.	.	.	.	.	.	.	.	.	.	.	.	C	.	.	.	.	.	.	.	.	.	.
	**643**	A	.	.	.	.	.	.	.	.	.	.	.	G	.	.	.	.	.	.	.	.	.	.	.	.	.	.	.	.	.	.	.	.	.
	**649**	T	.	.	.	.	.	.	.	.	C	.	.	.	.	.	.	.	.	.	.	.	.	.	.	.	.	.	.	.	.	.	.	.	G
	**673**	C	T	.	T	.	T	T	T	.	.	T	T	T	T	T	T	.	.	.	T	T	T	T	T	T	T	.	.	.	.	.	.	.	.
	**679**	T	.	.	.	.	.	.	.	.	.	.	.	.	.	.	.	.	C	.	.	.	.	.	.	.	.	.	.	.	.	.	.	.	C
	**691**	C	.	.	.	.	.	.	.	.	.	.	.	.	.	.	.	.	.	.	.	.	.	.	.	.	.	.	.	.	.	T	.	.	.
	**712**	A	G	.	G	G	G	G	G	.	G	G	G	G	G	G	G	.	G	.	G	G	G	G	G	G	G	.	.	.	.	.	.	.	G
	**721**	C	.	.	.	.	.	.	.	.	G	.	.	.	.	.	.	.	.	.	.	.	.	.	.	.	.	.	.	.	.	.	.	.	.
	**728**	T	.	.	.	.	.	.	.	.	.	.	.	.	.	.	.	.	.	.	.	.	.	.	.	G	G	.	.	.	.	.	.	.	.
	**736**	C	T	.	T	T	T	T	T	.	T	T	T	T	T	T	T	.	.	.	T	T	T	T	T	T	T	.	.	.	.	.	.	.	.
	**742**	C	T	.	T	T	T	T	T	.	T	T	T	T	T	T	T	.	T	.	T	.	T	T	T	T	T	.	.	.	.	.	.	.	T
	**745**	T	C	.	C	C	C	C	C	.	C	C	C	C	C	C	C	.	C	.	C	C	C	C	C	C	C	.	.	.	.	.	.	.	C
	**752**	G	.	.	.	.	.	.	.	.	.	.	.	.	.	.	.	.	.	.	.	.	.	.	.	.	.	.	.	A	.	.	.	.	A
	**757**	T	.	.	.	.	.	.	.	.	.	.	.	.	C	.	.	.	.	.	.	.	.	.	.	.	C	.	.	.	.	.	.	.	.
	**763**	A	.	.	.	.	.	.	.	G	.	.	.	.	.	.	.	G	.	.	.	.	.	.	.	.	.	G	.	.	.	.	.	.	.
	**772**	G	.	.	.	.	.	.	.	.	.	.	.	.	.	A	.	.	A	.	.	.	A	.	.	.	.	.	.	.	.	.	.	.	A
	**778**	T	.	.	.	.	.	.	.	.	.	.	.	.	.	C	.	.	.	.	.	.	.	.	.	.	.	.	.	.	.	.	.	.	.
	**781**	T	.	.	.	.	.	.	.	.	.	.	.	.	.	.	.	.	.	.	.	.	.	.	.	.	.	.	.	.	.	C	.	.	C
	**787**	C	T	.	T	T	T	T	T	.	T	T	T	T	T	T	T	.	T	.	T	T	T	T	T	T	T	.	.	.	.	.	.	.	T
	**793**	T	.	.	.	.	.	.	.	.	.	.	.	.	.	.	.	.	C	.	.	.	.	.	.	.	.	.	.	.	.	.	.	.	C
	**n**	3	5	1	7	1	1	1	6	1	1	2	2	1	4	1	9	1	1	3	1	4	4	2	1	1	1	4	3	1	3	2	1	1	1

Table 2 presents sequence variation across 570 base pairs of the mitochondrial cytochrome b and control region, that define 34 haplotypes of the reticulated python. Variable base pairs only shown (see text for NCBI GenBank accession numbers).

**Fig 2:**
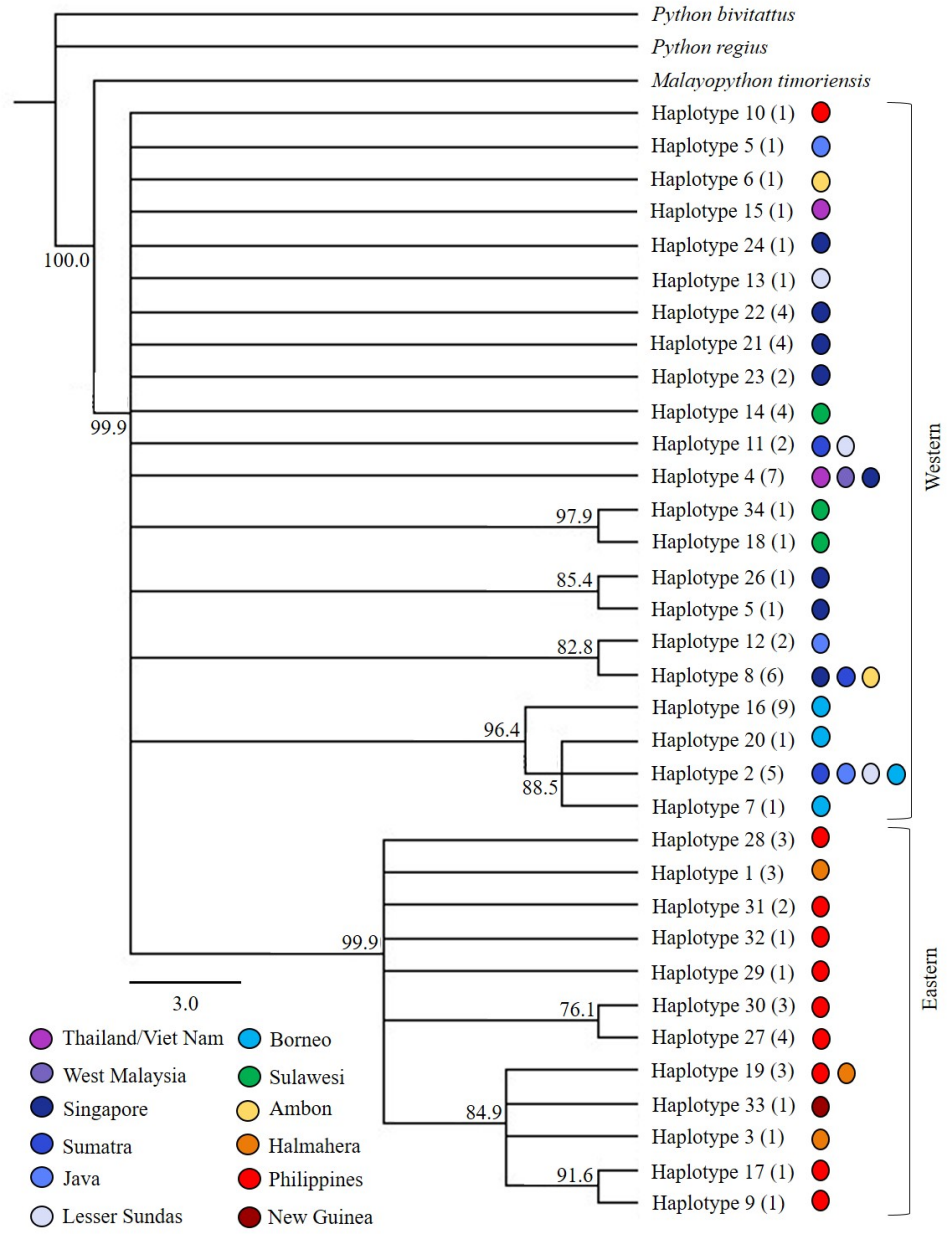
Phylogenetic analysis. Majority rule consensus tree for Bayesian inference of phylogeographic relationships among mitochondrial haplotypes inferred across the range of *M*. *reticulatus* (the bootstrap ML consensus tree resolved the same topology with respect to all well supported nodes). The tree was rooted with two more distantly related members of the genus *Python* and one more closely related member of *Malayopython* [22] and nodal support is provided as Bayesian posterior probabilities. The number of individuals per haplotype is given in parenthesis and coloured shapes indicate sampling location.

Both Bayesian and ML inference produced similarly unresolved tree topologies (Fig 2) with strong ML bootstrap support and posterior Bayesian support for five localised haplotype clades, but no support for branching order among these clades. These haplotype groups included moderate to strongly supported clades of haplotypes from (1) Sulawesi, (2) Singapore, (3) Java/Ambon/Sumatra/Singapore (4) Borneo/Sumatra/Lesser Sundas, and (5) the Philippines. Although there was moderate to strong support for the monophyly of *M*. *reticulatus* and five well-supported but unresolved clades, 12 unique haplotypes exhibited no statistically supported affinities to each other or the five aforementioned haplotype clades. The addition of more distantly related outgroup species (*P*. *bivitattus* and *P*. *regius*) did not resolve the branching order among clades. Except for a single haplotype (Haplotype 10), haplotypes in the Philippines and Halmahera formed a distinct monophyletic clade which fell into a basal polytomy with the remaining haplotypes. Haplotypes from Borneo (haplotypes 2, 7, 16 and 20) also clustered independently although one sequence (Haplotype 2) was shared with Java, Sumatra and the Lesser Sundas. Two haplotypes found only on Sulawesi (Haplotype 18 and 34) demonstrate a high level of sequence identity to each other (99.3%) and a lower sequence identity to the remaining haplotypes (95.5% and 94.6%, respectively), including Haplotype 14 which was also found on Sulawesi. It is possible these divergent haplotypes represent samples of the subspecies *M*. *reticulatus saputrai*, a distinct population of only known from Selayar Island and South Sulawesi [34]. Pairwise estimates of evolutionary divergence found within either the Philippines (0.7%–1.2%) or Singapore/Sumatra/Java/Borneo (1.2%–1.6%) were significantly lower than comparisons made between these two groups (4.4%–6.2%). The exception was Haplotype 10 which was more divergent from haplotypes in the Philippines (4.8%–5.4%). Tamura-Nei estimates of divergence between groups indicate a higher level between the Philippines and Sulawesi (5.5%), of the Philippines and Singapore (5.2%) than between Singapore and Sulawesi (4.4%).

**Fig 3:**
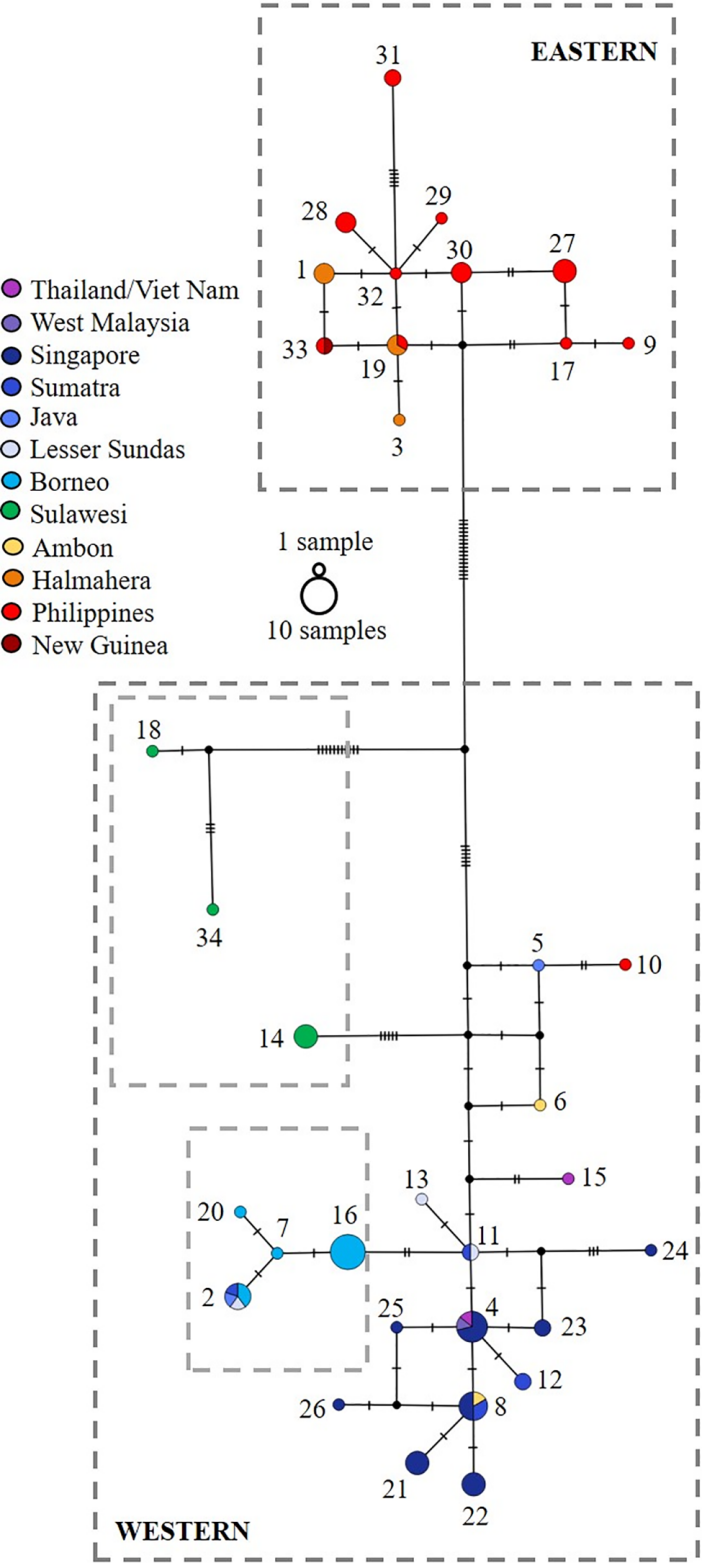
Median-joining network. Network of mitochondrial haplotypes. Each circle represents a unique DNA sequence and its frequency and geographic identity are denoted by the circle size and colour. Hatch marks represent 1 nucleotide substitution along the 570 bp sequence and dotted lines delineate the phylogenetically-defined groups (see text for discussion).

The median joining network identifies two geographically distinct groups of haplotypes (Fig 3). Fifteen percent of haplotypes are shared across multiple island locations (the Philippines being counted as a single location); most of the remaining haplotypes were found at only one island location (Fig 1). A total of 20 haplotypes were present across Thailand, Viet Nam, West Malaysia, Singapore, Sumatra, Java, Lesser Sundas, Borneo, Sulawesi and Ambon (in this study termed the ‘Western’ haplotype group). Bornean haplotypes clustered hierarchically within the Western haplotype group. Only one haplotype was shared with other locations and the most frequent haplotype (Haplotype 16) was only found on Borneo, where it represents 69% of individuals. Although not forming a distinct cluster in the network (they did form a strongly supported phylogenetic couplet in the phylogeographic analysis), haplotypes found on Sulawesi exhibit higher levels of differentiation than other Western haplotypes. Twelve haplotypes were present among individuals from the Philippines, Halmahera and a single sample erroneously labelled as ‘New Guinea’ (termed the Eastern haplotype group). Despite its geographic proximity to Ambon and Sulawesi, samples from Halmahera were more similar to those from the Philippines, with one haplotype shared between both locations (Haplotype19). Haplotype16 occurred at the highest frequency (n = 9) within the Eastern group and was only present across the Philippines. Within both Western and Eastern haplotype groups, haplotypes typically differed by 1–5 substitutions from their nearest neighbour. The mean within-group distance ranged from 0.6% (Eastern group) to 1.0% (Western group), with 5.5% divergence between the two. The probability of the clades being chosen via a random coalescent process was p < 0.05, suggesting that the division may represent natural, geographically endemic genetic variation, possible warranting formal taxonomic recognition. Median joining networks compiled using only the short sequence fragments (i.e. before concatenation) retained the two main haplotype groups (Western and Eastern) but resolution within each cluster was largely lost as the number of base pairs analysed was reduced.

When haplotypes were grouped as either ‘Western’ or ‘Eastern’, between-group differences accounted for 83.5% (p < 0.001) of the observed variation with a global AMOVA Φ_ST_ of 0.86 (p < 0.000001). All pairwise Φ_ST_ estimates between Singapore, Borneo, Sumatra, Halmahera and the Philippines, were significantly different, rejecting the null hypothesis of a single, panmictic population (or possibly, taxonomic entity). Overall haplotype diversity was H_d_ = 0.962 (± 0.008), with both the Western and Eastern groups showing comparable levels of this genetic diversity when estimated independently (Table 3). Haplotype and nucleotide diversities were highest for the Philippines (H_d_ = 0.915, π = 0.0104), then for Singapore (H_d_ = 0.876, π = 0.0032), Borneo (H_d_ = 0.526, π = 0.0015), and finally Sulawesi (H_d_ = 0.500, π = 0.0008). Haplotype and nucleotide diversity for the remaining insular populations could not be estimated due to the small sample sizes (n < 4).

**Table 3:** Population genetic summary statistics.

Haplotype group	Location	n	H	H_R_ (±SD)	π	S
**Western**	Thailand/Viet Nam	2	2	1.000 (0.500)	0.0063	5
	Peninsula Malaysia	1	1	N/A	N/A	N/A
	Singapore	21	8	0.876 (0.037)	0.0032	10
	Sumatra	6	4	0.867 (0.129)	0.0044	6
	Java	2	2	1.000 (0.500)	0.0155	10
	Lesser Sunda Islands	3	3	1.000 (0.272)	0.0052	5
	Borneo	13	4	0.526 (0.153)	0.0015	3
	Sulawesi^a^	4	2	0.500 (0.265)	0.0008	1
	Ambon	2	2	1.000 (0.500)	0.0086	5
	*Overall West*	*54*	*20*	*0*.*935 (0*.*016)*^b^	*0*.*0074*	*25*
**Eastern**	Halmahera	6	3	0.700 (0.048)	0.0027	3
	Philippines	18	10	0.915 (0.041)	0.0104	35
	New guinea	1	1	N/A	N/A	N/A
	*Overall East*	*25*	*12*	*0*.*931 (0*.*025)*^b^	*0*.*0007*	*38*

Table 3 contains population genetic summary statistics describing genetic diversity as a function of geographic sampling area.

^a^ Excluding two putative *Malayopython r*. *saputrai* individuals

^b^ Haplotype diversities rather than haplotype richness provided

## Discussion

Resolving the geographic distribution of the naturally occurring genetic variation of *Malayopython reticulatus* is an ongoing issue consisting of several distinct challenges. In addition to being shaped by the evolutionary diversification of the lineage, in response to the dynamic geographical template representing major landmasses in this realm [81,82], the contemporary geographical range of *M*. *reticulatus* has been influenced by several factors including its exceptional dispersal behaviour and ability to cross large stretches of water [30], environmental conditions [83], and anthropogenic translocation [31]. The latter issue has been highlighted by [84]who notes ‘*the reticulated python … is carried around as a food animal and rat-catcher’* and ‘*some of its occurrences on Wallacean islands’* are *‘possibly … due to human agency*’ (see comments [14,85]). Trade dynamics of *M*. *reticulatus* seem likely to have contributed to the introduction of non-native populations, and to have likewise facilitated the potential for genetic homogenisation between what would otherwise have been isolated populations.

Despite the potential for confounding factors, we revealed significant structure among mitochondrial haplotypes across widely distributed Southeast Asian populations of *M*. *reticulatus*. The 34 haplotypes resolved across the species expansive geographical range were structured between two broad geographic realms. The first comprised landmasses on the Sunda Shelf (continental Southeast Asia, Sumatra, Java, Borneo), the Lesser Sundas, and the oceanic islands of Sulawesi, Ambon and Palawan of the Philippines; the second included only the Philippines and Halmahera. In the context of this study, hese two geographic regions have been termed the Western and Eastern haplotype groups, respectively; the distinction reflects the dominant geographic location of the haplotypes found, although the ‘Western’ group is nested within the paraphyletic ‘Eastern’ group. Although mitochondrial haplotypes within the Western haplotype group showed a high degree of similarity to previously published *M*. *reticulatus* sequences (98%–99%), those found in the Eastern group appear more divergent (94%–96% sequence similarity). This level of divergence has previously been reported for three subspecies of *Python curtus* that were elevated to distinct species [52]; each subspecies contained less than 1% sequence divergence within the taxon, between 3% and 8.9% divergence between the sub-species, and 10%–12.4% sequence divergence from *P*. *curtus* sister species, *M*. *reticulatus*.

The shared ancestry of the land-bridge islands of the Sunda Shelf (Sumatra, Java and Borneo) with continental Southeast Asia is a likely result of the region’s paleogeographic setting [29,86], which was characterised by dramatically lowered sea levels and expanded continental land area during the Pleistocene glacial maxima [4,81,87–89]). Of the three islands, Sumatra and Java remain geographically proximal to both each other and to continental Southeast Asia. Despite the rising sea level which separated Sumatra and Java from the mainland, surface currents between the land masses are presumed not to have been strong enough to act as a barrier to the dispersal of *M*. *reticulatus* as demonstrated by the colonisation of Krakatau Island [30]. Active marine dispersal from Java to Sumatra and vice versa could be facilitated by the circulation of sea surface currents triggered by monsoon winds, i.e. December–February south-eastern currents from Sumatra to Java, then June–August north-western currents from Java to Sumatra [90,91]. The species may therefore have crossed marine barriers as has been reported for the estuarine crocodile (*Crocodylus porosus*) [92], however, it is also noted that the ‘*human agency on boats is highly significant’* with regard to the dispersal of terrestrial vertebrates in the Sunda Strait [93]. Conversely, individuals from Borneo form a nested cluster within the Western haplotype group and share only a single haplotype with other locations in this group. This difference could be evidence of an effective barrier to dispersal imposed by the islands greater distance from continental Southeast Asia, Sumatra or Java but the lack of resolution of branching order within this clade prevents confident interpretation of these possibilities.

**Fig 4:**
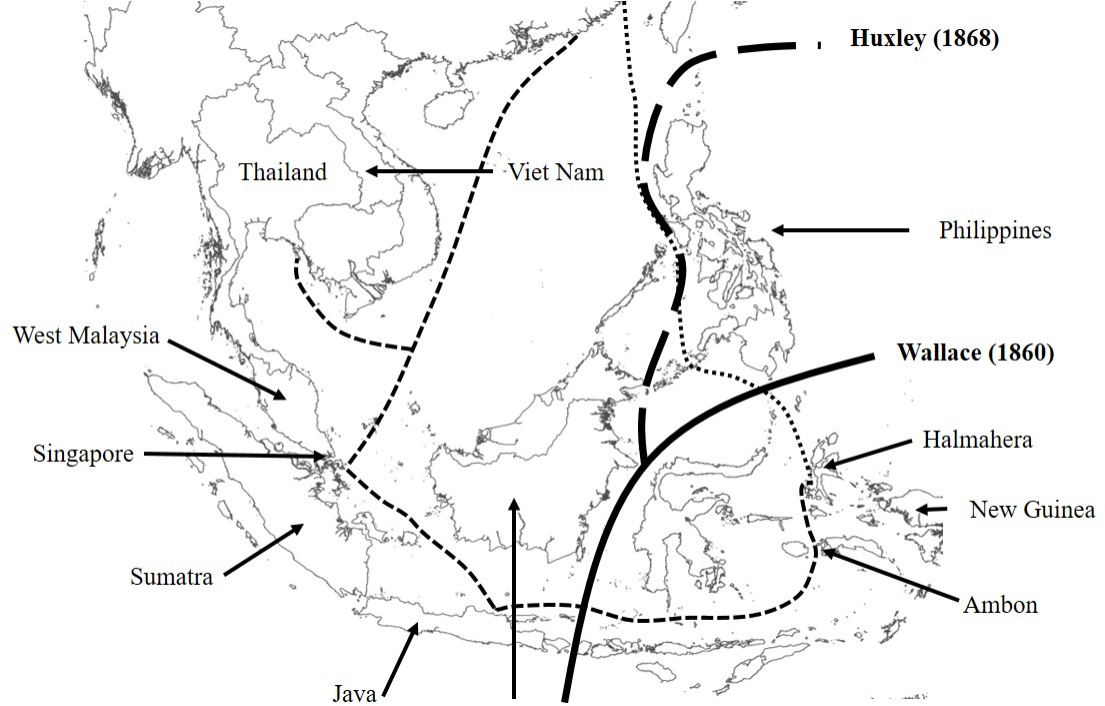
Biogeographic realms. Chinese trade routes to the North Moluccas. Dotted line indicates the eastern route used during the Yuan period (1279–1368). The dashed line indicates the route used by Chinese Merchants connecting Chinese overseas settlements to northern Java likely used during the Ming period (1368–1644). Faunal boundaries are indicated by the Wallace line [101] and Huxley's line [94]. Map revised after Ptak 1992 [102].

Interestingly, [85] surmised that *M*. *reticulatus* may not be native to the Philippines, and that human introductions might be responsible and explain the existence of the species in this archipelago. Divergence between individuals from the Philippines and members of the Western haplotype group (continental Southeast Asia, Sumatra, Java, Borneo, Lesser Sundas, and Sulawesi) is demarcated by Huxley’s modification of Wallace’s line, a theoretical, biogeographic boundary summarizing the eastern edge of the Sunda Shelf (Fig 4). Huxley’s modification of Wallace’s line separates the Philippines archipelago from the Sunda Shelf [94], with the ‘exception’ of Palawan which has been variably classified or associated with the Sunda Shelf [81,95–98]). This theoretical barrier corresponds to the distinction between the Eastern and Western haplotype groups described here, and the presence of the Palawan haplotype (Haplotype 10) in the Western (Sunda Shelf) group is an interesting example of a large vertebrate, distributed according to the view that Palawn is a final extension of Sundaland [97]. The Eastern versus Western pattern of divergence elucidated here has previously been documented in a variety of taxa but is far from universal in terrestrial vertebrates [81,97,99,100].

Northern Halmahera is part of an arc system, which is shared by the Philippines and northern New Guinea [82,103]. The islands now reveal similar distribution patterns of e.g., insects and rodents [104], with Halmahera located at the southern Philippine trench [105–107]. According to [108] the ‘east Halmahera-east Mindanao terrane’ is considered as an ‘eastward continuation of a Papuan arc complex’, termed as the Melanesian arc. This biogeographic affinity might explain the haplotype lineage found on Halmahera, that appears closely related to the Philippines; similar southern Mindanao biogeographic relationships have previously been inferred to involve Sulawesi [82,100,109]. This however, does not account for the divergence seen between Halmahera and Ambon which are both geographically close and located on the same biogeographic arc.

Geographical variants of mitochondrial DNA found on Ambon were more closely related to those present in Singapore and on Sumatra than nearby Halmahera. As part of the Seram Island Group (including the islands of the Outer Banda Arc, i.e. Seram, Ambon, Boano, Kelang, Maniapa, Harukuku and Saparua, see [110]), Ambon Island does have endemic species such as the python *Morelia clastolepis* [111] and monitor lizard *Varanus cerambonensis* [112], whereas other species are thought to have been introduced to Ambon e.g., marsupials, such as the northern common cuscus (*Phalanger orientalis*) and the common spotted cuscus (*Spilocuscus maculatus*) [113]. Historic anthropogenic movement of *M*. *reticulatus* individuals may account for the discordance between haplotype and geographic proximity across the Wallacea region as according to [31], *M*. *reticulatus* was also introduced to Ambon. Chinese trade routes that passed the Moluccas and Chinese overseas settlements in northern Java were in use during the Ming period and as the only Asian producers of cloves during this time, vessels would likely have visited the Moluccas for this commodity ([102], see Fig 4), and in particular, Ambon due to its accessibility along the shipping route. This may well have led to the introduction of *M*. *reticulatus* to Ambon following use aboard their ships as rat-catchers and food [102].

Although less divergent than the Philippines, and tentatively considered part of the Western haplotype group, Sulawesi shares no haplotypes with other group members. Representative of the Wallacean biogeographic faunal region and separated from the Sunda Shelf by deep underwater channels, divergence between faunal assemblages either side of Wallace’s line is well documented and a number of endemic species have been identified on Sulawesi, often further restricted to within one of seven areas of endemism [96,109]. A recent study also suggests that the population *Malayopython r*. *jampeanus* is genetically more closely related to the nominate form *M*. *r*. *reticulatus*, than to the geographical closer located *M*. *reticulatus saputrai* [34], suggesting genetic exchange between the Lesser Sundas and some of the Selayar Islands in the Flores Sea [114]. During the late Cretaceous, Borneo was still connected to western Sulawesi [87,115], thus enabling the exchange of terrestrial organisms; however, the formation of the Makassar Strait between both islands isolated terrestrial biota, explaining the high level of endemism on Sulawesi today (e.g., [116]). The Makassar Strait flow is considered a strong (sub-) surface current [117] that might prevent dispersal of *M*. *reticulatus* between both islands and explain the genetic distinction of Sulawesi haplotypes.

Despite the major tectonic collision events that occurred ca. 25 Million years ago, the current distribution patterns of terrestrial organisms in Southeast Asia were likely only formed during the last one Million years [29,86]. Formation of the world’s two largest archipelagos, Indonesia and the Philippines have, in part, resulted from very different tectonic processes [107]. Although major landmasses of the Greater Sundas and Palawan Island have been placed on the margin of the Eurasian plate, on the Sunda Shelf, most islands of the Philippines were paleotransported from the southeast [4,106]. Although the distribution of mitochondrial haplotypes for *M*. *reticulatus* spatially coincides with biogeographic expectations derived from the distribution of land and sea [86] and the geological mechanisms responsible for the formation of the archipelago [81], there are insufficient data to infer the exact number of colonisation events or the order in which they might have occurred [12]. Furthermore, comparison with haplotype sequences previously resolved for *M*. *reticulatus* is prevented by a paucity of equivalent data with associated locality information in Genbank. Nevertheless, the current dataset represents a step forward in that it is by far the most geographically comprehensive for *M*. *reticulatus*, Importantly, the phylogeographic structure inferred here suggests the existence of naturally occurring, properly documented genetic variation across the species distributional range. Although the sample size here is not conducive to fine-scale population genetic analysis, structuring within each of the main clades would be anticipated with the use of higher resolution markers, sampled from throughout the genome.

*Malayopython reticulatus* is currently managed as a single taxonomic unit across its range, and there is no consideration for genetic differences between harvested populations. Even though major countries of origin and export of *M*. *reticulatus* skins (Malaysia and Indonesia) have national/provincial harvest quotas [20], this management system is difficult to verify in terms of transparency and traceability [118]. There is no tool available to authenticate the likely origin of a skin except from the information included on the CITES exports permits, and listing the country of export does not automatically infer the country of origin. Illegal cross-border trade is known to exist, undoubtedly allowing unscrupulous traders to ‘circumvent national quotas’ [118], and loopholes in export reporting mechanisms have been repeatedly outlined in earlier studies [7,20,41,119,120]. In 2016, the provincial harvest quota was highest for South Sulawesi, followed by North Sumatra and West Kalimantan (Borneo) (see Table 4). The need to investigate the impact of regional harvest has been highlighted for the populations *M*. *reticulatus saputrai* and *M*. *r*. *jampeanus* [34]; however, trade in both is on-going with the former, to date, outnumbering provincial quotas set for Sumatra and Kalimantan (Anon. in litt. to Auliya; 22 Nov. 2016; see Table 4). Although there is no information available to address the impact that regional trade may have on the sustainability of different, taxonomically distinct, range restricted, populations of *M*. *reticulatus*, specific management tools are warranted to permit the identification of python skin origin, to ensure harvest quotas are adhered to, in order to maintain the viability of genetically distinct populations.

**Table 4:** Regional harvest quotas for the reticulated python.

Purpose	Island	Province	Harvest Quota
**Pet**	Kalimantan	West Kalimantan	1,100
** **	Sumatra	Bengkulu	400
** **		Lampung	200
** **		North Sumatra	1,300
** **		South Sumatra	300
** **		***Sub total***	***3*,*300***
**Skins**	Kalimantan	Central Kalimantan	15,600
** **		East Kalimantan	18,500
** **		South Kalimantan	11,000
** **		West Kalimantan	23,000
** **	Maluku Islands	Maluku	1,200
** **	Sulawesi	Central Sulawesi	5,000
** **		South Sulawesi	29,400
** **	Sumatra	Aceh	5,000
** **		Bengkulu	8,000
** **		Jambi	10,000
** **		Lampung	5,000
** **		North Sumatra	20,000
** **		Riau	65,000
** **		South Sumatra	13,000
** **		West Sumatra	5,000
** **	West Nusa Tenggara	West Nusa Tenggara	500
** **		***Sub total***	***176*,*700***
**Total**	** **	** **	**180,000**

Table 4 contains regional harvest quotas of 2016 for the reticulated python in Indonesia. Source: Decision of Director General, Directorate General Ecosystem and Natural Resources Conservation, SK. 283/KSDAE-SET/2015.

Despite uncertainty of the impact current harvest levels will have, particularly on ecological and long term on genetic variability, no precautionary measures have been established to prevent local declines and extinctions. [121] provided substantial biological and ecological information on the species harvested on the island of Sumatra. Although their study indicated apparent sustainability, the authors also note that ‘far more information will be needed before we can confidently assess sustainable levels of offtake of Indonesian pythons’, and the same conclusions were drawn within a study published 17 years later [122]. Both studies provide regional-based results (Sumatra) and the latter authors conclude that ‘*the harvest appears to be sustainable*’, ignoring the entire issue of trade dynamics (legal and illegal within a country and between countries), and thus genetic variation as a result of local adaptation. Therefore, the identification of sustainability indices will be required (see [123]). The phylogeographic inference presented here suggests that geographically distinct clades (in particular, the Philippines and continental Southeast Asia, but also Borneo) warrant separate consideration as Evolutionary Significant Units. Furthermore, the level of divergence estimated for populations in the Philippines would also seem to justify a taxonomic review, and it is conceivable that phylogenetically distinct species may be present. Divergent, evolutionary distinct lineages should be taken into consideration when establishing harvest quotas to avoid over-exploitation and the erosion of intra-specific genetic variation [11]. This requires a thorough understanding of the fine scale species structure and the source/skin dynamics of populations at different scales [124].

Because genetic divergence, lineage isolation, and even speciation are not necessarily accompanied by morphological divergence (see [125,126]), the use of DNA-based tools is a logical development for verifying the origin of traded python skins. Skins that are destined for incorporation to the luxury goods industry are heavily treated with a cocktail of chemicals, and so it is necessary to ensure that the same genetic signals can be detected in processed skins as in untreated ones before developing forensic tests and databases. Chemically treated skins can yield low concentrations of heavily degraded and fragmented DNA [127] and therefore, it is necessary to ensure that any DNA-based test devised be feasible for use with poor quality samples, from which only degraded DNA may be extracted. Although phylogeographic analysis of shorter mitochondrial sequence fragments did not retain the same number of haplotypes that were evident when using concatenated sequences, it did continue to distinguish the Western and Eastern haplotype groups, as well as the Sulawesi population. This demonstrates that the sequence data may be used to assign individuals of ‘unknown origin’ [11], back to either Western or Eastern genotypes. This result suggests that heavily processed samples of ‘unknown origin’ within the python skin trade (where amplification is restricted to small fragments of DNA; [128]) could be localised to geographic origin by comparison to the data presented here.

## Conclusions and recommendations

Although the results presented here offer a very provisional insight to the genetic structure of *Malayopython reticulatus* across the species contemporary range, they offer an encouraging conservation genetics baseline that justifies implementing a precautionary approach [129] to population management, and further consideration and investigation is necessary.

If genetically distinct populations of *Malayopython reticulatus* are to be continually exploited for the skin trade, management strategies should not solely be tailored to obtaining the maximum economic yield. Populations should instead be managed following an adaptive management scheme to ensure their long-term sustainability (see [130]). Among the complex geography of continental and insular Southeast Asia, trade dynamics currently outbalance sustainable measures [3,18], and thus effective management practises for preventing local over-exploitation are not established to address illegal trade activities and trace origins of sourced populations [20,41,131]

Genetic variation is fundamental for species conservation [96]. To safeguard genetically distinct populations, prevent genetic erosion and retain their ability to undergo evolutionary change, it is important to manage populations in such a way that they do not fall under a viable threshold [132]. Based on the results of this work, the following conclusions and recommendations are offered:

1. Whilst caution is to be exercised in inferring taxonomic divisions within the current dataset due to the limited availability of quality reference samples, the genetic distinctiveness of the Philippine population suggests that the status of *M*. *reticulatus* within this archipelago warrants further investigation and possible review.
2. Distinct genetic structure across the ecoregions and faunal regions suggests that variation in heritage should be incorporated into the implementation of regional management schemes (also see [55]), and may provide a basis for the development of regional skin traceability systems.
3. To regulate trade dynamics, wild harvest rates and exploitation levels require regular monitoring of the status of resource population(s) to reduce uncertainties.
4. Further fine scale genetic analysis is also warranted to delineate local genetic partitions, and the potential application of adaptive genetic markers to establish conservation units should be considered (see [133]).
5. To encourage and foster scientific collaborative networks with the countries of origin to permit construction of comprehensive, reference sample databases on which to base the development of robust traceability protocols.

## Supporting information

S1 Table Details of samples included in this study. The table contains information about samples included in this study along with the corresponding haplotypes inferred from sequence variation across a mitochondrial cytochrome b fragment.
(PDF)
