# Supplementary material for: Phylogeography of the reticulated python (Malayopython reticulatus ssp.): Conservation implications for the worlds’ most traded snake species
Source: PLoS One. 2017 Aug 17;12(8):e0182049. doi: 10.1371/journal.pone.0182049 (PMC5560690; doi:10.1371/journal.pone.0182049)
Supplement: S1 Table — The table contains information about samples included in this study along with the corresponding haplotypes inferred from sequence variation across a mitochondrial cytochrome b fragment. (PDF) [file pone.0182049.s001.pdf]

Original source	Sample type	Contributor sample reference	Country	Haplotype
The Senckenberg Museum Frankfurt	Tissue	1a / 16735	Halmahera	Haplotype 3
	Muscle	7 / 23296	Lesser Sundas Islands	Haplotype 2
Natural History Museum Vienna	Embryo	13:1 / 2360:1	Thailand	Haplotype 4
Naturalis, Leiden	Muscle	3 / RMNH.RENA. 36809	Ambon	Haplotype 6
Natural History Museum Vienna	Muscle	15:1 / 15580:1	Borneo	Haplotype 7
	Muscle	2 / 15590	Sumatra	Haplotype 8
	Muscle	3 / 13400:12	Philippines	Haplotype 9
	Muscle	4:1 / 13395	Palawan	Haplotype 10
	Muscle	7 / 13400:3	Singapore	Haplotype 4
	Muscle	9:1 / 15577:1	Sumatra	Haplotype 11
Zoological Museum Berlin, ZMB	Muscle	19 / 30901	Sumatra	Haplotype 12
	Muscle	10 / 33877	Sumbawa	Haplotype 13
Zoological Research Museum Alexander Koenig (ZFMK), Bonn.	Muscle	1 / 73479	Java	Haplotype 5
	Muscle	2 / 84234	Sulawesi	Haplotype 14
	Muscle	3 / 73478	Viet Nam	Haplotype 15
	Muscle	5 / 70208	Borneo	Haplotype 16
	Muscle	6 / 82822	Sulawesi	Haplotype 14
	Muscle	7 / 82823	Sulawesi	Haplotype 14
	Muscle	8 / 70126	Borneo	Haplotype 16
	Muscle	9 / 70128	Borneo	Haplotype 16
	Tissue	ZFMK81824	Borneo	Haplotype 16
	Tissue	ZFMK81805	Borneo	Haplotype 2
	Tissue	ZFMK81827	Borneo	Haplotype 16
	Tissue	ZFMK81797	Borneo	Haplotype 16
	Tissue	ZFMK81814	Borneo	Haplotype 16
	Tissue	ZFMK81813	Borneo	Haplotype 2
	Tissue	ZFMK81801	Borneo	Haplotype 16
Zoological Museum Hamburg	Muscle	6 / 9782	West Malaysia	Haplotype 4
	Muscle	7 / 9783	Philippines	Haplotype 17
Naturalis, Leiden	Muscle	2 / RMNH.RENA.41333	Sulawesi	Haplotype 18
	Muscle	4 / RMNH.RENA. 36810	Ambon	Haplotype 8
The Senckenberg Museum Frankfurt	Muscle	1b / 16736	Halmahera	Haplotype 19
	Muscle	5 / 16751	Borneo	Haplotype 20
	Muscle	6 / 16752	Sumatra	Haplotype 12
	Muscle	8 / 23297	Lesser Sundas	Haplotype 11

Original source	Sample type	Contributor sample reference	Country	Haplotype
Singapore Zoological Gardens	Blood	1/0770-0BBC/375	Singapore	Haplotype 21
	Blood	2/0769-1714/345	Singapore	Haplotype 4
	Blood	3/071A-18EE/352	Singapore	Haplotype 22
	Blood	4/0769-0A2E/381	Singapore	Haplotype 8
	Blood	5/0769-1844/372	Singapore	Haplotype 22
	Blood	6/0769-13CC/357	Singapore	Haplotype 4
	Blood	7/0770-09A9/366	Singapore	Haplotype 23
	Blood	8/0769-195E/349	Singapore	Haplotype 23
	Blood	9/074F-CBF1/339	Singapore	Haplotype 22
	Blood	10/0769-1A37/362	Singapore	Haplotype 21
	Blood	11/0770-0EBA/367	Singapore	Haplotype 22
	Blood	12/0770-B03A/360	Singapore	Haplotype 21
	Blood	13/0740-AA84/359	Singapore	Haplotype 8
	Blood	14/074F-BE43/343	Singapore	Haplotype 24
	Blood	15/0770-C1F8/368	Singapore	Haplotype 25
	Blood	16/0769-1BA7/378	Singapore	Haplotype 26
	Blood	17/0683-61B6/355	Singapore	Haplotype 4
	Blood	18/0769-1B00/373	Singapore	Haplotype 8
	Blood	19/0769-1999/376	Singapore	Haplotype 4
	Blood	20/0770-0E3E/353	Singapore	Haplotype 21
University of Kansas Biodiversity Institute (KU) & National Museum of the Philippines (PNM)	Tissue	RMB 3658, Deposited at PNM	Philippines	Haplotype 27
	Tissue	KU 307759	Philippines	Haplotype 28
	Tissue	KU 305171	Philippines	Haplotype 28
	Tissue	PNM/CMNH H1299	Philippines	Haplotype 29
	Tissue	KU 310867	Philippines	Haplotype 30
	Tissue	KU 315207	Philippines	Haplotype 31
	Tissue	KU 315208	Philippines	Haplotype 32
	Tissue	KU 335267	Philippines	Haplotype 28
	Tissue	KU 335266	Philippines	Haplotype 27
	Tissue	ACD 6347, Deposited at PNM	Philippines	Haplotype 30
	Tissue	KU 14696	Philippines	Haplotype 27
	Tissue	ACD 7241, Deposited at PNM	Philippines	Haplotype 30
	Tissue	KU 336060	Philippines	Haplotype 31
	Tissue	RMB 4522, Deposited at PNM	Philippines	Haplotype 27
Muséum National d'Histoire Naturelle, Paris	Tissue	MNHN_RA_0.5730(=1881.0023)	Philippines	Haplotype 19
	Tissue	MNHN_RA_1891.0064	Borneo	Haplotype 16
	Tissue	MNHN_RA_0.5922(=1882.0006)	New Guinea	Haplotype 33

Original source	Sample type	Contributor sample reference	Country	Haplotype
Breeder	Tissue	1_F	Halmahera	Haplotype 19
	Tissue	2_M	Halmahera	Haplotype 1
	Tissue	3_Un	Sulawesi	Haplotype 34
	Tissue	4_Un	Gebe Island	Haplotype 1
Breeder	Skin	10 /	Halmahera	Haplotype 1
	Skin	14 /	Sumatra	Haplotype 2
	Skin	11 /	Java	Haplotype 2
Breeder	shed skin	1	Sulawesi	Haplotype 14
	shed skin	5	Sumatra	Haplotype 8
